# Supplementary material for: MetaQTL: a package of new computational methods for the meta-analysis of QTL mapping experiments
Source: BMC Bioinformatics. 2007 Feb 8;8:49. doi: 10.1186/1471-2105-8-49 (PMC1808479; doi:10.1186/1471-2105-8-49)
Supplement: Additional File 7 — MetaQTL Package : jar file and tutorial. This Zip archive contains both the MetaQTL JAR file and the files of the tutorial. [file 1471-2105-8-49-S7.zip › org.inra.metaqtl/doc/org/metaqtl/IBioGenomeProperties.html]

IBioGenomeProperties


|  |  |  |
| --- | --- | --- |
| **Overview** \| **Package** \| **Class** \| **Use** \| **Tree** \| **Deprecated** \| **Index** \| **Help** | |  |
| **PREV CLASS**   **NEXT CLASS** | **FRAMES**    **NO FRAMES**     **All Classes** |  |
| SUMMARY: NESTED \| FIELD \| CONSTR \| METHOD | DETAIL: FIELD \| CONSTR \| METHOD |  |


---


## org.metaqtl Interface IBioGenomeProperties

---

``` public interface IBioGenomeProperties ```

This interface defines both the keys and the
default values of properties which
can be handled by a org.thalia.bio.entity.IBioGenome.

---

| **Field Summary** | |
| --- | --- |
| `static java.lang.String` | `CROSS_SIZE`             The cross size key. |
| `static java.lang.String` | `CROSS_TYPE`             The cross type key. |
| `static java.lang.String` | `MAPPING_CROSS`             The key for mapping cross. |
| `static java.lang.String` | `MAPPING_EXPANSION`             The key for mapping expansion status. |
| `static java.lang.String` | `MAPPING_FUNCTION`             The key for mapping function. |
| `static java.lang.String` | `MAPPING_FUNCTION_HALDANE`             The code for Haldane mapping function. |
| `static java.lang.String` | `MAPPING_FUNCTION_KOSAMBI`             The code for Kosambi mapping function. |
| `static java.lang.String` | `MAPPING_UNIT`             The mapping unit key. |
| `static java.lang.String` | `MAPPING_UNIT_CM`             The code for centi-Morgan unit. |
| `static java.lang.String` | `MAPPING_UNIT_M`             The code for Morgan unit. |

**Field Detail**

### CROSS\_SIZE

```
static final java.lang.String CROSS_SIZE
```

:   The cross size key.

    **See Also:**: Constant Field Values

---


### CROSS\_TYPE

```
static final java.lang.String CROSS_TYPE
```

:   The cross type key.

    **See Also:**: Constant Field Values

---


### MAPPING\_UNIT

```
static final java.lang.String MAPPING_UNIT
```

:   The mapping unit key.

    **See Also:**: Constant Field Values

---


### MAPPING\_UNIT\_M

```
static final java.lang.String MAPPING_UNIT_M
```

:   The code for Morgan unit.

    **See Also:**: Constant Field Values

---


### MAPPING\_UNIT\_CM

```
static final java.lang.String MAPPING_UNIT_CM
```

:   The code for centi-Morgan unit.

    **See Also:**: Constant Field Values

---


### MAPPING\_FUNCTION

```
static final java.lang.String MAPPING_FUNCTION
```

:   The key for mapping function.

    **See Also:**: Constant Field Values

---


### MAPPING\_FUNCTION\_HALDANE

```
static final java.lang.String MAPPING_FUNCTION_HALDANE
```

:   The code for Haldane mapping function.

    **See Also:**: Constant Field Values

---


### MAPPING\_FUNCTION\_KOSAMBI

```
static final java.lang.String MAPPING_FUNCTION_KOSAMBI
```

:   The code for Kosambi mapping function.

    **See Also:**: Constant Field Values

---


### MAPPING\_EXPANSION

```
static final java.lang.String MAPPING_EXPANSION
```

:   The key for mapping expansion status.

    **See Also:**: Constant Field Values

---


### MAPPING\_CROSS

```
static final java.lang.String MAPPING_CROSS
```

:   The key for mapping cross.

    **See Also:**: Constant Field Values


---


|  |  |  |
| --- | --- | --- |
| **Overview** \| **Package** \| **Class** \| **Use** \| **Tree** \| **Deprecated** \| **Index** \| **Help** | |  |
| **PREV CLASS**   **NEXT CLASS** | **FRAMES**    **NO FRAMES**     **All Classes** |  |
| SUMMARY: NESTED \| FIELD \| CONSTR \| METHOD | DETAIL: FIELD \| CONSTR \| METHOD |  |


---
